# Prevalence and Characteristics of Persistent Symptoms in Children During the COVID-19 Pandemic: Evidence From a Household Cohort Study in England and Wales

**DOI:** 10.1097/INF.0000000000003715

**Published:** 2022-10-21

**Authors:** Faith Miller, Dr Vincent Nguyen, Annalan MD Navaratnam, Madhumita Shrotri, Jana Kovar, Andrew C Hayward, Ellen Fragaszy, Robert W Aldridge, Pia Hardelid

**Affiliations:** From the * Institute for Global Health, University College London, UK; † Institute for Health Informatics, University College London, UK; ‡ Great Ormond Street Institute of Child Health, University College London, UK.

**Keywords:** SARS-CoV-2, persistent symptoms, prevalence, long COVID, chronic fatigue

Following initial acute infection with severe acute respiratory syndrome coronavirus 2 (SARS-CoV-2), individuals may experience persistent, postacute symptoms, which can last for many months.^1^ Commonly referred to as “long COVID”, persistent symptoms following SARS-CoV-2 infection can affect multiple organs and body systems, including the heart, lungs, and immune system, ranging in severity from persistent anosmia, headaches, and fatigue to reduced lung function and myocardial inflammation.^2–4^ Persistent symptoms have been reported by patients with severe^5–6^ and mild acute SARS-CoV-2 infection.^7^ Compared with adults, children have been much less severely affected by COVID-19 disease; in Europe, 1.7% of COVID-19 hospital admissions during 2020 (before vaccination programs were rolled out) were in children.^8^ It remains unclear to what extent children are affected by persistent SARS-CoV-2-related symptoms.^9^

Understanding the frequency and nature of persistent symptoms following SARS-CoV-2 infection in children is crucial for understanding the severity of the infection and informing COVID-19 pediatric vaccination policy. Previous estimates of the prevalence of persistent symptoms (lasting >4 weeks) following SARS-CoV-2 infection have been inconsistent. Analysis of data from a UK symptom app indicated a 4.4% prevalence of persistent symptoms 4 weeks after infection.^10^ Prevalence in a UK-based national survey was first reported as 9.8-13.0% (depending on age group),^11^ but this was later revised downward to 3.3–4.6%.^12^ A large case-control study in the UK reported a prevalence of 66.5% three months after initial infection, notably higher than the proportion of children reporting symptoms at the time of infection.^13^ The estimated prevalence of persistent symptoms in children following a SARS-CoV-2-related hospital admission has generally been higher; ranging from 8% to 53%.^14–16^ Differences in case definition and follow-up length across different studies make robust prevalence estimation in different pediatric populations difficult.^9^ Further, health services would benefit from evidence on whether SARS-CoV-2 is more or less likely to cause persistent symptoms than other viral infections.

The objective of this study was to estimate the prevalence of persistent symptoms reported among all children, including those with a history of SARS-CoV-2 infection, between June 2020 and May 2021. We also aimed to identify risk factors associated with persistent symptoms during the COVID pandemic among all children participating in a large household-based community cohort study.

## MATERIALS AND METHODS

### Study Design

We used data from VirusWatch, a household cohort study conducted in England and Wales. Study design and recruitment are described elsewhere.^17^ Briefly, households were recruited starting in mid-June 2020 via methods including postal service, social media, and text messages. By May 2021, 53,136 individuals in 25,555 households had registered, in line with sample size calculations informed by previous experience.^17^ To participate, households required internet access, an email address, and for at least one household member to read and understand sufficient English for survey completion. Households with more than six individuals were excluded due to limitations in the Research Electronic Data Capture (REDCap) survey infrastructure. All household members provided informed consent, or assent for children aged 6-15 years old. Parents consented on behalf of children aged <6 years old. Participating households completed an online baseline questionnaire and were followed up using weekly surveys, monthly themed topic surveys, and linkage to the National Health Service (NHS) SARS-CoV-2 testing program in hospitals and the community. A subset of around 11,000 participants was included in the laboratory testing sub-cohort (see text, Supplemental Digital Content 1; http://links.lww.com/INF/E823), who provided blood samples using venipuncture (between September 2020-January 2021 and April-September 2021) or finger-prick (between October 2020-August 2021), as well as nasopharyngeal swab samples for PCR assay of SARS-CoV-2^17^ if they or their household contacts displayed a defined set of symptoms.

Mid-year population estimates by age, gender, and Index of Multiple Deprivation quintile (IMD; see below) were obtained from the Office for National Statistics (ONS) for comparison.^18,19^

### Study Population and Period

We defined persistent symptoms using data from weekly surveys and the February 2021 and May 2021 monthly surveys, which asked about persistent symptoms (the “persistent symptom” surveys; distributed on February 17 and May 26, 2021, respectively). For this analysis, we included all children aged ≤17 years at enrolment who had either (a) answered the question about persistent symptoms in the February or May monthly surveys, or (b) whose household had participated in at least 3 weekly surveys over 5 weeks, before April 21, 2021 (5 weeks before the May monthly survey). This was to ensure all included children would have a sufficient follow-up to be at risk of the outcome.

### Outcome

Table 1 summarizes the source of all variables included in our analysis. In the February and May monthly surveys, participants were asked whether they had experienced any new, persistent symptoms lasting four or more weeks. If they had, they could also report the nature of the symptoms, the extent to which symptoms affected daily activities, and the date of onset and resolution for the three most severe symptoms (Table 1).

**TABLE 1.** Description and Source of All Variables Included in Analysis

Variable Type	Variable	Source
Infection exposure	SARS-CoV-2 infection history	VirusWatch baseline survey *“Have you ever had a positive swab test for COVID-19?”*VirusWatch testing of symptomatic participants and contacts *Participants were asked to submit a nose/throat swab if they experienced 2 consecutive days of COVID-19-related symptoms, with household member testing in a subset of participants (see Supplementary file 1 for further detail).*VirusWatch laboratory testing sub-cohort; SARS-CoV-2 IgG testing *See supplemental file 1 for information on serology testing*Data Linkage to NHS Digital or SGSS *NHS Digital linked the VirusWatchcohort to SARS-CoV-2 tests collected in the community(the “Pillar 2” dataset) and hospitals (the Second Generation Surveillance System [SGSS] dataset*)
Outcome	Persistent symptoms	February 2021 and May 2021 monthly surveys *“In the last year (since February 2020) have any of the household members experienced any new symptoms that have lasted for four or more weeks even if these symptoms come and go, and that are not explained by something else (eg, pre-existing chronic illness or pregnancy)?” (response: yes/no*)Weekly surveys; Symptoms reported over ≥4 weeks *“Have you or anyone in the household had any of these symptoms in the past week?”(response: yes/no*)
	Persistent symptom types	February 2021 and May 2021 monthly surveys *“Please pick all of the new symptoms that you have experienced for four or more weeks. These are new symptoms that you experienced for four or more weeks in the last year and that are not explained by something else (e.g. pre-existing chronic illness or pregnancy)”*
	Persistent symptoms affecting daily activities	February 2021 and May 2021 monthly surveys *“Did your symptoms make it more difficult to:Go to/participate in work or education?*,*Concentrate on things (e.g. reading, watching TV)?*,*Take care of yourself (e.g. wash, dress, and feed yourself)?*,*Take care of others in the household?*,*Do necessary daily activities outside the house (e.g. shopping)?, orDo activities that you enjoy (e.g. hobbies)?” (response for each: Yes a lot, Yes a little, Not at all, N/A*)
	Start and end date of persistent symptoms	February 2021 and May 2021 monthly surveys *“Approximately when did this symptom first appear?”, “Is it still ongoing?”, and if not ongoing “Approximately when did this symptom first stop?”*Weekly surveys; Symptoms reported over ≥4 weeks *“Which days did you have symptoms?”*
Covariates	Age	Baseline survey *Calculated from date of birth*
	Gender	Baseline survey *“At birth you were described as?” (response: male, female, intersex, prefer not to say*)
	Socio-economic deprivation	Baseline survey *Indices of multiple deprivation (IMD) mapped to postcode at residence*
	Long-term conditions or medications	Baseline survey *“Has a doctor or other health professional ever told you that you have any of the following conditions?” (Select from list of long-term conditions, including “other”*)*“Which of the following medicines do you take?” (Select from list of medications*)

We defined “persistent symptoms” as a child having either answered yes to the above question in the February or May monthly surveys or reporting symptom episodes lasting four weeks or more through the weekly surveys. This definition was in line with the National Institute for Health and Care Excellence (NICE) guidance and published studies at the time of survey design.^7,9–11^ Symptom start and end dates were self-reported in the monthly and weekly surveys. If the information on persistent symptoms was derived from the weekly survey, the start date of the illness episode was used as the onset of persistent symptoms. Those answering “unsure” in either monthly survey (0.6% of those answering the question) were assumed to not experience persistent symptoms.

We coded persistent symptoms into groups used by NICE: respiratory, cardiovascular, generalized (including fatigue and fever), neurological (including cognitive impairment/“brain fog” and headache), gastrointestinal, psychiatric, ear, nose, and throat (ENT), dermatological or other symptoms.^20^

Those reporting persistent symptoms in the monthly surveys were asked if their persistent symptoms impacted regular activities (Table 1). Children were coded as their symptoms impacting daily activities if they answered “*yes, a lot*” or “*yes, a little*” to any of these.

### Exposure

History of SARS-CoV-2 infection was determined using (i) self-report at baseline, (ii) swabs administered by the VirusWatch survey (sent to symptomatic participants and their contacts^17^), (iii) presence of SARS-CoV-2 immunoglobulin G (IgG) through VirusWatch serology testing (as part of the VirusWatch laboratory testing sub-cohort), or (iv) linkage to NHS Digital data on results of SARS-CoV-2 tests collected by NHS community care (the “Pillar 2” dataset) and hospitals (the Second Generation Surveillance System [SGSS] dataset; Table 1).

If the estimated date of a positive swab was before or <10 days after persistent symptom onset, we assumed infection occurred before persistent symptoms. When children had positive swabs from multiple sources, the date of the first positive result was chosen. For children missing a swab date who reported using the weekly surveys, we estimated it to be the previous Monday. For those missing a swab date who reported using the baseline survey, we estimated it as the 15th of the reported month. For those missing a swab date who were IgG seropositive, we were not able to determine the date of infection, and we assumed infection occurred before the start of persistent symptoms.

### Covariates

Information on age, gender, socio-economic deprivation, and the presence of long-term conditions was obtained at baseline. Age was coded into three groups: <2, 2–11, and 12–17 years. The presence of a long-term condition was coded as a binary variable based on reports of children’s long-term conditions and medications from the baseline questionnaire. A small-area level indicator of socio-economic deprivation, the IMD (coded into quintiles), and region of residence were mapped to the household postcode.^21^ Gender was reported at baseline.

### Statistical Analyses

We compared the distribution of age, sex, region of residence, and IMD quintile in the study cohort with that of the resident population of children in England and Wales in 2019.^18,19^

We estimated the prevalence of persistent symptoms according to age group, sex, IMD quintile, presence of a long-term condition, and whether the child had a history of SARS-CoV-2 infection. We fitted mixed-effects logistic regression models for persistent symptom prevalence including these risk factors as independent variables *a priori*, and household ID as the random intercept. A substantial proportion of children had not reported their gender; therefore, we included a category of “missing gender” in the regression model. Throughout, statistical significance was defined as no overlap of 95% confidence intervals for compared parameters.

We estimated the median duration of persistent symptoms for children with a date of onset and completion for at least one symptom and calculated the proportion of children whose symptoms impacted daily activities. All analyses were carried out using Stata version 16 and RStudio version 3.4.3.

The study received ethical approval from the Hampstead NHS Health Research Authority Ethics Committee (20/HRA/2320). The use of SARS-CoV-2 test results data via NHS Digital was approved by the Health and Social Care Information Centre for this study (DARS-NIC-372269-N8D7Z-v1.6).

### Sensitivity Analyses

To check the robustness of our definition of persistent symptoms, we repeated analyses combining symptom episodes from the weekly reports which were 8 and ≤ 14 days apart to allow for symptoms to “come and go”. We further repeated analyses excluding children (a) whose gender was missing, or (b) who only had serological evidence of prior SARS-CoV-2 infection (as we were not able to confirm that infection occurred before symptom onset).

## RESULTS

We included 5032 children, 1729 of whom were in the laboratory testing subcohort (34.4%). Children 12–17 years old and those living in the East of England were overrepresented in the VirusWatch child cohort compared with mid-year population estimates. The child cohort was substantially less deprived than the population of children in England (see Table, Supplemental Digital Content 2, http://links.lww.com/INF/E824). 498 children reported at least one long-term condition (9.9%), 402 of whom (80.7%) reported having clinician-diagnosed asthma or using an inhaler (8.0% of the cohort).

A total of 1062 cohort children (21.1%) had evidence of past or present SARS-CoV-2 infection (see Table, Supplemental Digital Content 2, http://links.lww.com/INF/E824). Of these, 854 positive cases (80.4%) were identified using the NHS Pillar 2 testing data and 318 (29.9%) using SGSS. 28 (2.6%) children reported their infection status at baseline, 381 (35.9%) had a positive VirusWatch swab, and 101 (9.5%) were positive on serology. A total of 461 (43.4%) positive cases were identified using one method only, while 601 (56.6%) were identified using two or more methods.

The overall prevalence of persistent symptoms was 2.6% overall (129/5,032 children; 95% CI 2.1-3.0%), and 4.1% (43/1062 children; 95% CI, 2.9–5.4%; Table, Supplemental Digital Content 3, http://links.lww.com/INF/E825) in children who had a history of SARS-CoV-2 infection before persistent symptom onset. 66.7% of children with persistent symptoms (86/129) had no evidence of past SARS-CoV-2 infection.

Children with a history of SARS-CoV-2 infection were 1.8 times more likely to report persistent symptoms compared with children who did not (95% CI, 1.1–3.1; Table, Supplemental Digital Content 3, http://links.lww.com/INF/E825). Being a teenager or having a long-term condition significantly increased the odds of persistent symptoms. The point estimate of the odds ratio indicated that girls had an increased risk of experiencing persistent symptoms, but this was not statistically significant. The proportion of children reporting persistent symptoms did not appear to vary significantly by IMD quintile (see Table, Supplemental Digital Content 3, http://links.lww.com/INF/E825). Excluding children who had not reported their gender did not substantially change the association between SARS-CoV-2 infection status and persistent symptoms (see table, Supplemental Digital Content 2; http://links.lww.com/INF/E824). Sensitivity analysis excluding the 8 children who reported persistent symptoms and were only positive on serology (with no estimated date of infection) reduced the point estimate of the OR for SARS-CoV-2 infection status to 1.55 (95% CI, 0.91–2.63) (Supplemental Digital Content 2 (Table); http://links.lww.com/INF/E824). Combining symptom episodes 8 or ≤ 14 days apart did not substantially change the association between SARS-CoV-2 infection status and persistent symptoms (see Table, Supplemental Digital Content 3; http://links.lww.com/INF/E825).

**Figure 1.**
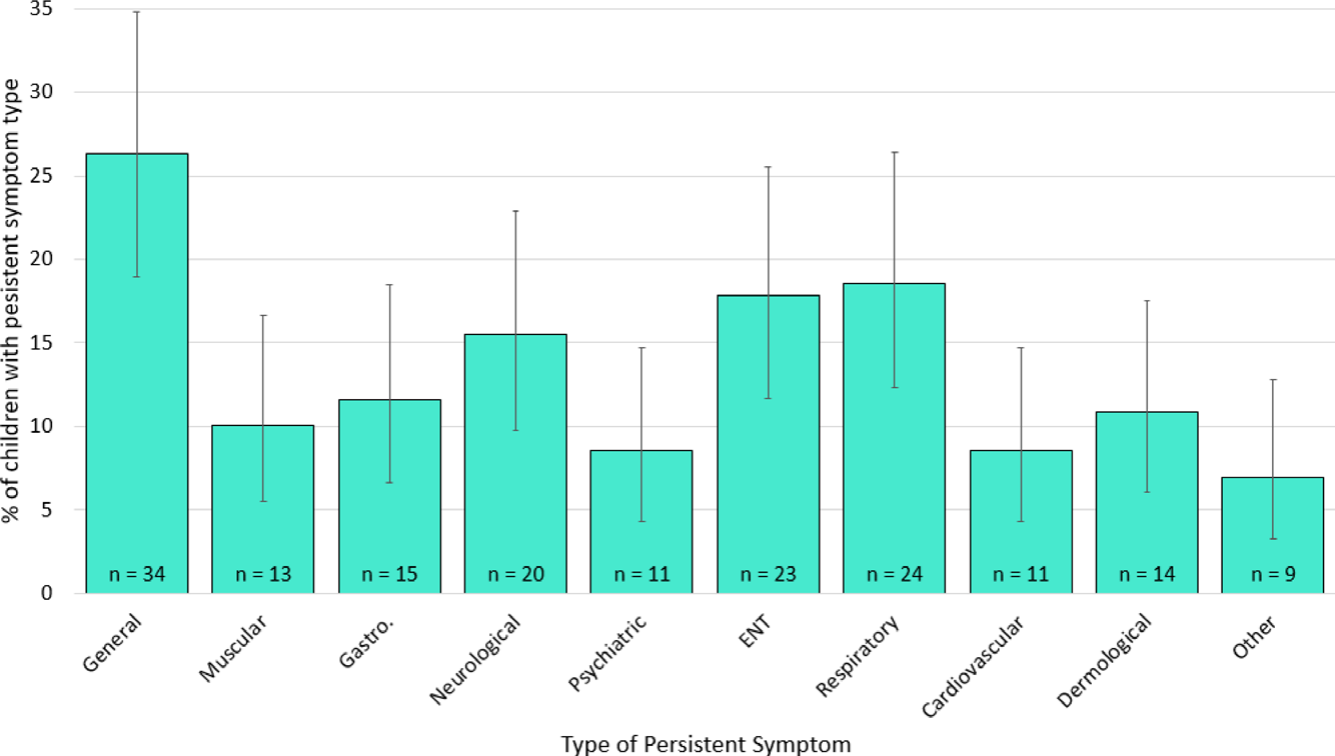
The proportion of children reporting each symptom type out of all children reporting persistent symptoms who also reported type of symptoms (*n* = 109), categorized according to the National Institute for Health and Care Excellence categorizations, with 95% confidence intervals. ENT, ear, nose, and throat; Gastro, gastro-intestinal.

Overall, the most commonly reported persistent symptom types were general, respiratory, and ENT (Fig. 1). There were no significant differences in the proportion of children reporting any of the symptom types according to SARS-CoV-2 infection status. For the three most commonly reported symptoms, 30.2% of children with infection history reported general symptoms (95% CI, 16.5–44.0%), compared with 24.4% of those without infection history (95% CI, 15.4–33.5%), 14.0% of children with infection history reported respiratory symptoms (95% CI 3.6–24.3%), compared with 21.0% of those without infection history (95% CI, 12.3–29.5%), and 14.0% of children with infection history reported ENT symptoms (95% CI, 3.6–24.3%), compared with 20.0% of those without infection history (95% CI, 11.4-–28.2%). 43.9% of children with a history of SARS-CoV-2 infection who experienced persistent symptoms (18/41) reported that these symptoms had an impact on regular activities. 46.1% of children without a history of SARS-CoV-2 infection (35/76) reported their persistent symptoms impacted regular activities.

The median duration of persistent symptoms was 33 days (interquartile range 30–74) for the 28 children who reported start and end dates of at least one symptom. For the 6 children with a history of SARS-CoV-2 infection with known start and end dates, the median duration of persistent symptoms was 32 (interquartile range 29–41).

## DISCUSSION

The prevalence of persistent symptoms lasting ≥4 weeks in children during the first 18 months of the COVID-19 pandemic was 2.6% overall, and 4.1% among children with a history of SARS-CoV-2 infection. Teenagers and children with long-term conditions were more likely to report persistent symptoms. 67% of children with persistent symptoms had no evidence of a history of SARS-CoV-2 infection.

### Strengths and Limitations

We used data from a large sample of children resident in England and Wales, representative of the general population of children in terms of age and sex. We were able to compare the prevalence of persistent symptoms in children with and without a history of SARS-CoV-2 infection. This is important to capture the wider health consequences of the pandemic on all children in the cohort, such as fatigue and low mood as a result of national lockdowns, and health consequences from lack of access to education, social activities, health services, and reduced physical activity.^22,23^ By collecting swabs, serology blood samples, and weekly reports of symptoms and test results, as well as linking to NHS testing data, we provide a more sensitive method of identifying children with a history of SARS-CoV-2 infection than swabs alone. Furthermore, the longitudinal design of the VirusWatch cohort study enabled us to capture the relapsing and remitting nature of persistent symptoms.

The prevalence of persistent symptoms was low in this cohort, meaning larger studies are required to assess risk factors for persistent symptoms in children related to SARS-CoV-2 infection in more detail. VirusWatch children were less socio-economically deprived than the general population, limiting generalizability. However, area-level socio-economic deprivation did not appear to be associated with persistent symptoms. Households with over six members, who did not speak or read sufficient English, or who did not have internet access were also not eligible for participation, further limiting the generalizability of findings. Since most children experiencing persistent symptoms did not report an end date, we were not able to assess time to symptom resolution for all children. However, current evidence indicates that most children recover after two months.^3,5^ Previous studies have used a definition of persistent symptoms following SARS-CoV-2 infection (“long COVID”) including symptoms ranging from 4 to 15 weeks, limiting comparison between studies.^10–12,24,25^ The World Health Organization has developed a clinical case definition for long COVID which includes symptoms lasting at least three months, but as our survey was designed before this was agreed this definition was not used.^26^

We were not able to compare the prevalence of persistent symptoms in children following SARS-CoV-2 infection to persistent symptoms following other respiratory infections. Chronic fatigue was reported by up to 13% of children after glandular fever/infectious mononucleosis (caused by Epstein–Barr virus).^27^ However, there is a dearth of evidence of multiple long-term symptoms following viral infections in children. Furthermore, social distancing measures have led to the suppression of many other respiratory viruses during 2020-2021,^28^ therefore the prevalence of persistent symptoms may be lower during this period than in previous years.

### Interpretation

Our estimate of the prevalence of persistent symptoms in children with a history of SARS-CoV-2 infection is similar to that reported via a UK symptom app^10^ and in a study of primary school children.^25^ Results from the UK-based CLoCK study,^29^ which includes a Delphi process to define long COVID in children,^30^ are considerably higher than our estimates, however, they limited their study to 11-17-year-olds, among whom persistent symptoms are higher.^24^ Further, CLoCK recruited 13% of approached participants with the specific aim of studying persistent symptom prevalence; participation bias may have led to a higher prevalence of reported persistent symptoms than reported in VirusWatch.^29^

The ONS has carried out several UK-based surveys which have established inconsistent estimates of the prevalence of persistent symptoms depending on data collection methods.^11,12^ Our estimate was lower than ONS estimates when relying on self-report,^11^ but similar to ONS estimates when following up children with a confirmed SARS-CoV-2 infection.^12^ As expected, the prevalence of persistent symptoms in children in VirusWatch appears lower than among children seen in secondary care.^5,16^ Nationally representative studies following up children hospitalized with SARS-CoV-2 infection, including suitable a control group such as children hospitalized for other infections, are required to assess symptom persistence in children with more severe COVID-19 disease. The most common persistent symptom among children in VirusWatch was fatigue, as reported elsewhere.^10,14^ As most episodes of persistent symptoms were ongoing at the time of the report, the median duration of symptoms reported here (33 days) is more than likely an underestimate.

We observed a higher prevalence of persistent symptoms among children who had a history of SARS-CoV-2 infection, in line with findings from the ONS survey and symptom app study, but in contrast to two serosurveys where children reporting symptoms did not know their infection status.^31,32^ Therefore, there is a risk of a reporting bias, in which the prevalence of persistent symptoms is overestimated among children with a history of SARS-CoV-2 infection status, as most children with a positive swab or serology knew their infection status.

## CONCLUSION

Our study contributes to a growing evidence base regarding the prevalence, risk factors, and characteristics of long COVID in children. To enable the assessment of risk factors and long-term impacts on health and education, further studies using consistent case definitions and control groups are urgently needed.^33^ Improved support for all children with persistent symptoms, particularly those with pre-existing long-term conditions, is required.

## Acknowledgements

(Members of the VirusWatch Collaborative include Susan Michie, Linda Wijlaars, Eleni Nastouli, Moira J Spyer, Ben Killingley, Ingemar Cox, Vasileios Lampos, Rachel A McKendry, Tao Cheng, Yunzhe Liu, Anne M Johnson, Jo Gibbs, Richard Gilson, Cyril Geismar, Sarah Beale, Isobel Braithwaite, Thomas E Byrne, Wing Lam Erica Fong, Parth Patel, Anna Aryee, Alison Rodger. We also wish to thank all VirusWatch participants for their support for this study)

## Supplementary Material


