# Supplementary material for: Prevalence and Characteristics of Persistent Symptoms in Children During the COVID-19 Pandemic: Evidence From a Household Cohort Study in England and Wales
Source: Pediatr Infect Dis J. 2022 Oct 21;41(12):979–84. doi: 10.1097/INF.0000000000003715 (PMC9645448; doi:10.1097/INF.0000000000003715)
Supplement: Supplementary file 1 [file inf-41-0979-s001.docx]

**Supplemental Digital Content 1.** Information on the laboratory testing sub-cohort of VirusWatch

**1. Virus Watch PCR testing**

Seventeen hundred twenty-nine children were included in the laboratory cohort (please see the study protocol^1^ for further details regarding the Virus Watch laboratory testing cohort). Participants in this sub-cohort were asked to submit nasopharyngeal swab specimens following certain symptom triggers suggestive of COVID-19 in themselves or a household member. Swab specimens were posted by the participants to the Francis Crick Institute, where they were tested for SARS-CoV-2 RNA via RT-PCR.

**2. Virus Watch serology testing**

Children in Virus Watch were invited to provide blood samples for SARS-CoV-2 serology testing either at a clinic, where venous blood samples were taken via phlebotomy, or through a rapid diagnostic test using a finger prick capillary blood sample.

2a. Clinic based blood sampling

A total of 10ml of whole blood were collected from children (≤15 years): by a member of the local research team with appropriate training and experience in phlebotomy.  Children were offered Emla cream 20min before blood draw. Samples were sent to the Francis Crick Institute, where they were tested for IgG antibodies against the S1-subunit of the Spike protein of SARS-CoV-2.2 Sampling was conducted between September 2020-January 2021 and April 2021-September 2021.

2b. Finger prick blood sampling

Finger prick capillary blood samples were collected via self-sampling (aided by a parent/guardian for children), and participants used a rapid diagnostic test (Fortress Diagnostics, Antrim, UK) to self-test for SARS-CoV-2 IgG and IgM against the Spike protein. Sampling was conducted between October 2020 and August 2021.

**References**

1. Hayward A, Fragaszy E, Kovar J, et al. Risk factors, symptom reporting, healthcare-seeking behaviour and adherence to public health guidance: protocol for Virus Watch, a prospective community cohort study. *BMJ Open* 2021;11(6):e048042. doi: 10.1136/bmjopen-2020-048042

2. Ng KW, Faulkner N, Cornish GH, et al. Preexisting and de novo humoral immunity to SARS-CoV-2 in humans. *Science* 2020;370(6522):1339-43. doi: 10.1126/science.abe1107
