# Supplementary material for: Prevalence and Characteristics of Persistent Symptoms in Children During the COVID-19 Pandemic: Evidence From a Household Cohort Study in England and Wales
Source: Pediatr Infect Dis J. 2022 Oct 21;41(12):979–84. doi: 10.1097/INF.0000000000003715 (PMC9645448; doi:10.1097/INF.0000000000003715)
Supplement: Supplementary file 2 [file inf-41-0979-s002.docx]

**SDC 2.** Characteristics of the VirusWatch Child Cohort and comparison with England & Wales population

|  | **VirusWatch child cohort**  *n = 5,032* | **England & Wales**  *n = 12,653,507* |
| --- | --- | --- |
| **Variable** | **Number (%)** | |
| **Age group** |  |  |
| <2 | 353 (7.02) | 1,325,800 (10.5) |
| 2-11 years | 2749 (54.63) | 7,321,164 (57.9) |
| 12-17 years | 1930 (38.35) | 4,006,543 (31.7) |
| **Gender** |  |  |
| Male | 2238 (44.48) | 6,489,021 (51.3) |
| Female | 2031 (40.36) | 6,164,486 (48.7) |
| Missing | 763 (15.16) | - |
| **Region** |  |  |
| East Midlands | 465 (9.24) | 1,002,649 (7.9) |
| East of England | 908 (18.04) | 1,346,457 (10.6) |
| London | 823 (16.36) | 2,032,427 (16.1) |
| North East | 222 (4.41) | 532,057 (4.2) |
| North West | 492 (9.78) | 1,563,460 (12.4) |
| South East | 890 (17.69) | 1,969,297 (15.6) |
| South West | 344 (6.84) | 1,107,477 (8.8) |
| Wales | 93 (1.85) | 629,939 (5.0) |
| West Midlands | 254 (5.05) | 1,299,803 (10.3) |
| Yorkshire & Humber | 241 (4.79) | 1,169,941 (9.2) |
| Missing | 300 (5.96) | - |
| **IMD Quintile*** |  |  |
| 1st quintile, most deprived | 480 (9.54) | 3,151,456 (23.7) |
| 2^nd^ | 730 (14.51) | 2,753,699 (20.7) |
| 3^rd^ | 923 (18.34) | 2,514,215 (18.9) |
| 4^th^ | 1133 (22.52) | 2,402,419 (18.1) |
| 5th quintile, least deprived | 1466 (29.13) | 2,460,532 (18.5) |
| Missing | 300 (5.96) | - |
| **Long term condition reported** |  |  |
| Yes | 498 (9.9) | - |
| **History of SARS-CoV-2 infection** |  |  |
| Yes** | 1062 (21.1) | - |
| **Persistent symptoms reported** |  |  |
| Yes | 129 (2.56) | - |
