# Supplementary material for: Prevalence and Characteristics of Persistent Symptoms in Children During the COVID-19 Pandemic: Evidence From a Household Cohort Study in England and Wales
Source: Pediatr Infect Dis J. 2022 Oct 21;41(12):979–84. doi: 10.1097/INF.0000000000003715 (PMC9645448; doi:10.1097/INF.0000000000003715)
Supplement: Supplementary file 4 [file inf-41-0979-s004.docx]

|  | a) Combining symptom episodes 8d apart | | b) Combining symptom episodes 14d apart | |
| --- | --- | --- | --- | --- |
|  | OR | 95% CI | OR | 95% CI |
| **Age group** |  |  |  |  |
| <2 years | 1.91 | (0.98 - 3.72) | 3.33 | (2.09 - 5.29) |
| 2-11 years | 1 | - | 1 | - |
| 12-17 years | 1.82 | (1.27 - 2.61) | 1.40 | (1.01 - 1.92) |
| **Gender** |  |  |  |  |
| Male | 1 | - | 1 | - |
| Female | 0.24 | (0.07 - 0.77) | 0.23 | (0.08 - 0.63) |
| Missing | 1.35 | (0.96 - 1.91) | 1.33 | (0.99 - 1.79) |
| **IMD Quintile** |  |  |  |  |
| 1st quintile (most deprived) | 0.73 | (0.37 - 1.42) | 0.85 | (0.48 - 1.50) |
| 2nd | 0.93 | (0.55 - 1.55) | 0.94 | (0.59 - 1.49) |
| 3rd | 0.92 | (0.57 - 1.47) | 1.08 | (0.72 - 1.62) |
| 4th | 0.71 | (0.44 - 1.14) | 0.86 | (0.57 - 1.30) |
| 5th quintile (least deprived) | 1 | - | 1 | - |
| **Any long-term condition reported** |  |  |  |  |
| No | 1 | - | 1 | - |
| Yes | 2.13 | (1.39 - 3.26) | 1.99 | (1.34 - 2.94) |
| **History of SARS-CoV-2 infection before symptom onset**^◦^ |  |  |  |  |
| No | 1 | - | 1 | - |
| Yes | 1.70 | (1.18 - 2.45) | 1.64 | (1.18 - 2.27) |

**Supplemental Digital Content 4.** Adjusted odds ratios for children experiencing persistent symptoms from a random effects logistic regression model when a) combining multiple symptom episodes which were 8 days apart, and b) combining multiple symptom episodes which were 14 days apart. ^◦^Before or up to 10 days after onset of persistent symptoms

*Abbreviations: aOR, adjusted odds ratios; IMD, Index of Multiple Deprivation; SARS-CoV-2, severe acute respiratory syndrome coronavirus 2*
