# Supplementary material for: Prevalence and Characteristics of Persistent Symptoms in Children During the COVID-19 Pandemic: Evidence From a Household Cohort Study in England and Wales
Source: Pediatr Infect Dis J. 2022 Oct 21;41(12):979–84. doi: 10.1097/INF.0000000000003715 (PMC9645448; doi:10.1097/INF.0000000000003715)
Supplement: Supplementary file 1 [file inf-41-0979-s002.docx]

**Supplemental Digital Content 2.** Adjusted odds ratios for children experiencing persistent symptoms from a random effects logistic regression model when a) excluding children who were identified through serology only and whose blood was taken after symptom onset and we assumed infection occurred before persistent symptom onset, and b) excluding children with a missing gender. ^◦^Before or up to 10 days after onset of persistent symptoms ᶧ When date of serology was post persistent symptom onset

|  | a) Excluding serology only ᶧ |  | b) Excluding missing gender |  |
| --- | --- | --- | --- | --- |
|  | OR | 95% CI | OR | 95% CI |
| **Age group** |  |  |  |  |
| <2 years | 1.74 | (0.67 - 4.49) | 1.69 | (0.78 - 3.66) |
| 2-11 years | 1 | - | 1 | - |
| 12-17 years | 2.38 | (1.43 - 3.96) | 2.16 | (1.47 - 3.18) |
| **Gender** |  |  |  |  |
| Male | 1 | - | 1 | - |
| Female | 1.48 | (0.93 - 2.36) | 1.35 | (0.94 - 1.94) |
| Missing | 0.24 | (0.06 - 0.94) | - | - |
| **IMD Quintile** |  |  |  |  |
| 1st quintile (most deprived) | 0.65 | (0.25 - 1.70) | 0.73 | (0.36 - 1.47) |
| 2nd | 0.85 | (0.39 - 1.82) | 0.93 | (0.54 - 1.59) |
| 3rd | 0.85 | (0.42 - 1.73) | 0.82 | (0.50 - 1.37) |
| 4th | 0.57 | (0.28 - 1.16) | 0.66 | (0.40 - 1.09) |
| 5th quintile (least deprived) | 1 | - | 1 | - |
| **Any long-term condition reported** |  |  |  |  |
| No | 1 | - | 1 | - |
| Yes | 2.53 | (1.34 - 4.79) | 2.03 | (1.30 - 3.17) |
| **History of SARS-CoV-2 infection before symptom onset**^◦^ |  |  |  |  |
| No | 1 | - | 1 | - |
| Yes | 1.55 | (0.91 - 2.63) | 1.73 | (1.18 - 2.55) |

*Abbreviations: aOR, adjusted odds ratios; IMD, Index of Multiple Deprivation; SARS-CoV-2, severe acute respiratory syndrome coronavirus 2*
